# Beyond the Amazon: vector surveillance and emerging Oropouche virus in South America

**DOI:** 10.1093/ve/veaf081

**Published:** 2025-10-15

**Authors:** Ruth Dálety da Silva Brito, Jéssica Pires Farias, Alexander Birbrair, Luís Carlos de Souza Ferreira, Paloma Oliveira Vidal, Jaime Henrique Amorim

**Affiliations:** Western Bahia Virology Institute, Center of Biological Sciences and Health, Federal University of Western Bahia, Barreiras, BA, Brazil; Vaccine Development Laboratory, Department of Microbiology, Biomedical Sciences Institute, University of São Paulo, Sao Paulo, SP, Brazil; Department of Dermatology, School of Medicine and Public Health, University of Wisconsin-Madison, Madison, Wisconsin, United States; Vaccine Development Laboratory, Department of Microbiology, Biomedical Sciences Institute, University of São Paulo, Sao Paulo, SP, Brazil; Western Bahia Virology Institute, Center of Biological Sciences and Health, Federal University of Western Bahia, Barreiras, BA, Brazil; Western Bahia Virology Institute, Center of Biological Sciences and Health, Federal University of Western Bahia, Barreiras, BA, Brazil

**To the Editor**,

We commend the recent study by Vieira *et al.* (Vieira et al. 2024) for the innovative approach of integrating xeno-monitoring and metatranscriptomic sequencing to investigate arboviral diversity. This innovative approach not only revealed diverse co-circulating arboviruses in Australia but also exemplifies how next-generation surveillance can outperform conventional methods in ecologically complex regions. Tropical areas in South and Central America, especially the Amazon basin and surrounding biomes, share similar conditions for arboviral endemicity, vector heterogeneity, and underdiagnosis due to symptom overlap following infection with different co-circulating viruses (Files et al. 2022, Bai et al. 2025). These findings highlight the potential of genomic surveillance tools in arbovirus-endemic regions.

**Figure 1:**
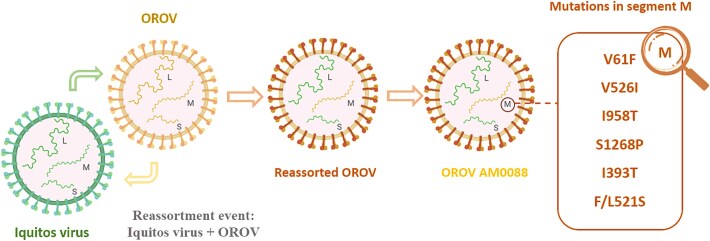
Schematic representation of the recent evolutionary events leading to the emergence of epidemic OROV in Brazil, in which a genomic reassortment likely occurred between 2022 and early 2023, involving the replacement of the small (S) and large (L) segments of the prototype OROV genome by those of Iquitos virus (IQTV), while retaining the medium (M) segment from the classical OROV lineage, and in mid-2023, a novel epidemic strain—AM0088—was identified, carrying multiple non-synonymous substitutions in the M segment, and was associated with the unprecedented OROV epidemic in Brazil in 2024, marked by increased viral replication in mammalian cells, larger and earlier plaque formation, and substantial reduction in neutralization by antibodies induced by prior OROV infection (Scachetti et al. 2025).

Of particular relevance stand neglected arboviruses such as the Oropouche virus (OROV), which is currently expanding its geographic and epidemiological footprint across the Americas (Gutierrez et al. 2020, Moreira et al. 2024). While historically endemic to the Amazon basin, OROV recently caused outbreaks in previously non-endemic areas of Brazil, including the northeast (e.g. Bahia State), the southeast (e.g. Espírito Santo State), and the south (e.g. Santa Catarina State), as well as in neighbouring countries, such as Peru and Panama (Moreira et al. 2024, Bai et al. 2025). These outbreaks were geographically distinct across multiple Brazilian states and neighbouring countries, with partially overlapping time windows during 2024. Recent genomic surveillance revealed that latest outbreaks are associated with a novel reassortant virus variant, whose small and large segments are more closely related to Iquitos virus, a related Orthobunyavirus, while the medium segment retains similarity to the OROV prototype strain (Gutierrez et al. 2020, Moreira et al. 2024). This reassortment event is accompanied by the emergence of non-synonymous mutations across different genomic segments (Fig. 1). In the M segment, substitutions V61F (in the Gn envelope glycoprotein), V526I, I958T, S1268P, and F/L521S (in the Gc envelope glycoprotein), as well as I393T in the nonstructural protein NSm, were identified. Mutations in the Gc envelope glycoprotein, which functions as the viral antireceptor, may influence host range and susceptibility by altering virus–receptor interactions. In the L segment, which encodes the RNA-dependent RNA polymerase (RdRp), substitutions such as I758V, I1942V, H476Y, E847G, and R346K were identified. These evolutionary and geographic events are summarized in Table 1. These amino acid changes may have phenotypic consequences, potentially affecting viral fitness, replication efficiency, and virulence (Moreira et al. 2024, Silva Júnior et al. 2024, Scachetti et al. 2025). Despite these alarming developments, OROV remains a neglected pathogen, with widespread underreporting due to limited molecular diagnostic capacity and lack of systematic vector surveillance programs (de Souza Luna et al. 2017, Bai et al. 2025). In addition, it is important to emphasize that in most places, vector surveillance is focused on mosquitoes and not midges. This diagnostic gap hinders early detection, impairs outbreak response, and masks the true disease burden, particularly in rural and peri-urban settings.

**Table 1:** Summary of evolutionary events and spillover geography of OROV

Year/period	Evolutionary event	Genomic segment/protein	Geography (spillover location)	Reference
2022–23	Reassortment with IQTV	S & L segments replaced; M retained	Amazon Basin (Brazil)	Gutierrez et al. 2020, Moreira et al. 2024
Mid-2023	Identification of epidemic strain AM0088	M segment with multiple non-synonymous substitutions	Brazil (Amazon to coastal expansion)	Scachetti et al. 2025
2024	Emergence of unprecedented epidemic	V61F, V526I, I958T, S1268P, F/L521S (Gn/Gc); I393T (NSm); RdRp substitutions	Brazil: Bahia, Espírito Santo, Santa Catarina; Peru; Panama	Moreira et al. 2024, Silva Júnior et al. 2024, Scachetti et al. 2025

Notes: Events are aligned with genomic surveillance reports cited in the main text. Terminology harmonized with Fig. 1 for consistency. Spillover refers to reported outbreaks in previously non-endemic regions.

Effective surveillance of OROV must consider the diversity of vectors involved in the transmission cycle. Species such as *Aedes serratus*, *Culex quinquefasciatus*, *Coquillettidia venezuelensis*, and *Mansonia venezuelensis* have been found naturally infected in the field, indicating their participation in the virus’s sylvatic cycle. Laboratory studies further confirmed that mosquitoes, including *A. serratus*, *Aedes scapularis*, *Aedes albopictus*, *C. quinquefasciatus*, and *Psorophora ferox*, can be experimentally infected and are capable of transmitting OROV, with *C. quinquefasciatus* exhibiting the highest vector competence described to date. However, it is important to clarify that the actual epidemiological relevance of mosquitoes remains uncertain, and no species has yet demonstrated the consistent field-to-human transmission dynamics established for biting midges. Nevertheless, the biting midge *Culicoides paraensis* remains the primary vector historically associated with human outbreaks, with its competence firmly established through virus isolation in wild specimens and successful transmission to susceptible hosts under laboratory conditions (Bai et al. 2025). The possibility of expansion of vector range raises urgent questions about urban spillover and the risk of future outbreaks in densely populated regions. In this context, metatranscriptomic surveillance applied directly to field-collected vector pools—as implemented in the Australian study—offers a powerful, unbiased approach to detect emerging OROV variants, including reassortant variants and co-infections with other arboviruses. Such methods can uncover evolutionary trajectories, inter-species transmission events, and genomic signatures of adaptation that are otherwise missed by conventional virological assays (Gutierrez et al. 2020, Vieira et al. 2024). Integrating these techniques into routine vector monitoring would not only enhance early detection of OROV but also support more accurate modelling of its spatial dynamics and evolutionary potential.

The successful implementation of genomic arbovirus surveillance through xeno-monitoring and metatranscriptomics in Australia offers a timely and transferable model for Latin America, where arbovirus emergence is shaped by ecological pressures, human mobility, and climate change (Sah et al. 2024). We advocate for the urgent adaptation of such strategies to South American contexts, particularly in Brazil, where arboviruses like OROV, mayaro virus (MAYV), yellow fever virus (YFV), and others demonstrate the capacity to cause unexpected outbreaks through sylvatic-urban spillover events (Moreira et al. 2024, Bai et al. 2025). Establishing regional genomic surveillance networks—integrating public health laboratories, academic institutions, and field entomology teams—would not only enhance early warning capabilities but also foster data sharing and coordinated responses across national borders. This collaborative infrastructure is essential to detect, characterize, and contain emerging viral threats before they reach epidemic scale. In addition, broader impact modelling of metatranscriptomic implementation and comparative epidemiological insights across related arboviruses represent promising directions for future research. However, the implementation of such strategies in Brazil and neighbouring countries faces practical challenges. These include the high cost of sequencing, limited access to equipped laboratories, the need for specialized training, and barriers to data integration across institutions. Recognizing and addressing these constraints is essential for the success of genomic surveillance in the region. In addition, recent studies have emphasized that the resurgence of OROV poses a diagnostic challenge in the region (Cherem and Barçante 2025), with frequent misclassification as dengue, reinforcing the need for expanded genomic surveillance and improved differential diagnosis.
